# Corrosion Assessment of Steel Bars Used in Reinforced Concrete Structures by Means of Eddy Current Testing

**DOI:** 10.3390/s16010015

**Published:** 2015-12-24

**Authors:** Naasson P. de Alcantara, Felipe M. da Silva, Mateus T. Guimarães, Matheus D. Pereira

**Affiliations:** Department of Electrical Engineering, São Paulo State University—Unesp, Bauru 17033-360, Brazil; felipe.matos.silva@gmail.com (F.M.S.); ra131010761@feb.unesp.br (M.T.G.); matheus.dantas.ismart@gmail.com (M.D.P.)

**Keywords:** reinforced concrete structures, corrosion process, nondestructive testing, eddy current testing, accelerated corrosion techniques

## Abstract

This paper presents a theoretical and experimental study on the use of Eddy Current Testing (ECT) to evaluate corrosion processes in steel bars used in reinforced concrete structures. The paper presents the mathematical basis of the ECT sensor built by the authors; followed by a finite element analysis. The results obtained in the simulations are compared with those obtained in experimental tests performed by the authors. Effective resistances and inductances; voltage drops and phase angles of wound coil are calculated using both; simulated and experimental data; and demonstrate a strong correlation. The production of samples of corroded steel bars; by using an impressed current technique is also presented. The authors performed experimental tests in the laboratory using handmade sensors; and the corroded samples. In the tests four gauges; with five levels of loss-of-mass references for each one were used. The results are analyzed in the light of the loss-of-mass and show a strong linear behavior for the analyzed parameters. The conclusions emphasize the feasibility of the proposed technique and highlight opportunities for future works.

## 1. Introduction

Reinforced concrete structures are, nowadays, the main construction element in most countries. However, despite flexibility and other construction advantages, reinforced concrete presents some problems that need constant monitoring. One of the main problems that fall within this scope is the process of corrosion of the reinforcements. In fact, the corrosion process dramatically affects the long-term performance of reinforced concrete structures, because it affects the flexural strength, deformation behavior, ductility, bond strength and mode of failure of the structures (El Maaddawy and Soudky [1]).

Corrosion of steel in concrete structures is as an oxidation process, followed by the breakdown of the passive film of the steel, due to the entry of chloride ions or carbon dioxide. In the initial phase the corrosion crack doesn’t happen directly on the surface of the concrete structure, but only shows up when the corrosion product reaches its threshold value. In addition, after the appearance of corrosion cracks on the surface, the rate of corrosion increases significantly due to the increased inflow of chloride ions or carbon dioxide through the cracks. In conclusion, this acceleration of the corrosion process threatens the safety of reinforced concrete structure (Maruya *et al*. [2]).

**Figure 1:**
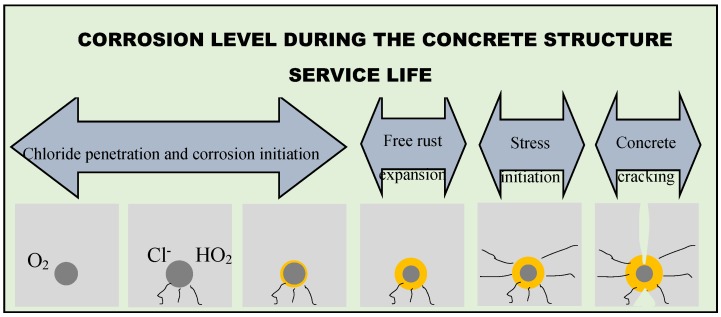
Schematic illustration of the various steps of the concrete deterioration due to the corrosion of the reinforcement (adapted from [3]).

As a result of the corrosion process, the corrosion product volume is two to six times greater than the original volume of the steel bar; so, this volume expansion causes cracking and spalling of the concrete cover, reduction of the cross-sectional area of the reinforcing steel volume, beyond that of the already mentioned negative effects. Consequently, the reinforcement corrosion reduces the load-carrying capacity of the structure, and brittle failure may occur without warning. According to Roqueta *et al*. [3], quoting Arndt and Jalinoos [4], there are six phases in the concrete corrosion process for nondestructive monitoring of the service life of a concrete structure, as depicted in Figure 1.

The aim of this paper is to present an experimental study on the use of Eddy Current Testing (ECT) to evaluate corrosion processes in the steel bars used in reinforced concrete structures. The following sections will present a survey of the techniques used to evaluate the corrosion in reinforced structures, the mathematical basis, and details of the ECT sensors built for the tests, computational simulations and comparisons with experimental results, the production of corroded samples of steel bars, results of experimental tests and their analysis.

## 2. A very Brief Survey of the Corrosion Assessment of Reinforced Concrete Structures

### 2.1. Electrochemical Techniques 

Although, in general, visual inspection is the most common practice for the evaluation of the conservation status of reinforced concrete structures, it is an inappropriate choice for checking the existence of corrosion processes. Signs of damage, such as cracks and spalling, when they appear, are indicative of an extensive corrosion process, so it is desirable to monitor the corrosion process in the reinforcement, starung with the structure construction phase, by conducting periodic inspections, and keeping a record of data.

There are different methods to assess the reinforcement corrosion on existing structures such as: (1) open circuit potential measurements; (2) surface potential measurements; (3) linear polarization resistance measurements; (4) galvanostatic pulse transient methods; (5) electrochemical impedance spectroscopy; (6) harmonic analysis and (7) noise analysis. This is not an exhaustive list, but it reflects the most common methods studied and used in recent years. Their basic principles remain unchanged, but some new technological contributions have been added over time. Below there is a brief description of each cited method:

(1) A metal body in contact with the surrounding media develops an electric potential. In reinforced concrete structures, the concrete acts as an electrolyte, generating an electrostatic potential, which can vary from place to place, depending on the state of the concrete. The principle involved in the open circuit potential measurements is essentially the measurement of the corrosion potential of the rebar to a standard reference electrode. This is the most typical procedure for the routine inspection of reinforced concrete structures (Erdogdu *et al*. [5]).
(2) During the corrosion process, an electrical current flows through the concrete between the anodic and cathodic regions. Measurements of the difference of potential at the concrete surface detect this current flow. Surface potential measurements are a non-destructive test to identify anodic and cathodic regions in concrete structures and, indirectly, to detect corrosion processes of the reinforcement. Two reference electrodes are used for the measurements, and no electrical connection to the rebar is required. An electrode is held fixed on the structure in a symmetrical point. The other electrode, called moving electrode, is moved to the nodal points of a grid, along the structure. The measurements are done using a high-impedance voltmeter. A positive reading of the voltage represents an anodic area where corrosion is possible. The higher the potential difference between the anodic and cathodic areas, the higher is the probability of corrosion (Song and Saraswathy [6]).
(3) The unique electrochemical technique with quantitative ability regarding the corrosion rate is the so-called polarization resistance, R_p_. This technique is based on the application of a small electrical perturbation to the rebar by using a counter electrode and a reference electrode. If the electrical signal is uniformly distributed throughout the reinforcement, the ∆E/∆I ratio defines R_p_. The corrosion current, I_corr_, is inversely proportional to R_p_, or, I_corr_ = B/R_p_, where B is a constant. R_p_ can be measured employing direct current or alternating current techniques (Andrade and Alonso [7]).
(4) The galvanostatic pulse method is a transient polarization technique working in the time domain. A short time anodic current pulse is impressed galvanostatically on the reinforcement from a counterelectrode placed on the concrete surface. The reinforcement is polarized in the anodic direction compared to its free corrosion potential. A reference electrode records the resulting change of the electrochemical potential of the reinforcement. Applying a constant current to the system, an intermediate ohmic potential jumps, and a slight polarization of the rebars occur. Under the assumption that a simple Randles circuit describes the transient behavior of the rebars, the potential of the reinforcement, *V(t)*, at a given time t, can be expressed by an exponential expression, plus a constant resistance (Sathiyanarayanan *et al*. [8]).
(5) Measurement of the electrochemical impedance is done by imposing a sinusoidal voltage (or current) signal of small amplitude, and by measuring the response signal of voltage and current. The amplitudes and the phase difference between the two signals are then analyzed. The frequencies vary between 10^−5^ and 10^5^ Hz, and the amplitudes between 10 mV and 10 V (MacDonald *et al.* [9]).
(6) The harmonic analysis method is an extension of the impedance method. Its execution is faster and leads to results that are more straightforward than those of the electrochemical impedance method. This technique is carried out by imposing an A.C. voltage perturbation at a single frequency and measuring the A.C. current density, i_1_. Two higher harmonics i_2_ and i_3_ are also measured. The harmonic analysis uses the fact that the corroding interface acts as a rectifier, in that the second harmonic current response is not linear about the free corrosion potential (Vedalakshmi *et* *al.* [10]).
(7) In the electrochemical noise method, measurements of the spontaneous fluctuations of the corrosion potentials and currents, which are observed as electrically coupled pairs, are taken. This method is random in nature. The range of frequency is typically from 10^−3^ to 1.0 Hz. Typical amplitudes are of the order of µV to mV, for voltage, and from nA to µA, for current. Electrochemical noise is a low-cost nondestructive technique reasonably straightforward, although attention must be paid to avoid problems, such as instrument noise, extraneous noise, aliasing, and quantization (Sheng *et al*. [11]).

### 2.2. Electromagnetic Techniques 

The previous section presented some techniques for the identification of corrosion process in reinforced concrete structures, based on electrochemical phenomena. In this section, we present the use of techniques for rebar inspection, as well as to detect corrosion processes, based on electromagnetic phenomena. Of course, this is not a state-of-the-art review, but it will serve to contextualize the present work in this scenario.

Electromagnetic fields are classified in stationary fields, low-frequency varying fields, and high-frequency varying fields. These three types of electromagnetic fields are used to develop non-destructive technology (NDT) techniques to assess the reinforcement of concrete structures.

Makar and Desnoyer [12], and Wolf and Vogel [13] presented examples of the use of magnetic flux leakage (MFL) method, produced by magnetostatic fields, to detect failures in concrete rebars. In both papers, the MFL method was used to detect breaks in steel tendons of prestressed structures.

Eddy current testing (ECT) is the best known technique in the NDT area based on low-frequency electromagnetic fields. Shull [14] and Garcia-Martin *et al*. [15] have presented very well the principles of ECT. In the reinforced concrete inspection context, Rubinacci *et al*. [16] presented an example of the use of this technique. They used the ECT principles to develop a numerical model, based on the finite element method, to locate and identify the size of steel bars under the concrete. Alcantara [17], built differential electromagnetic sensors, produced dozens of reinforced concrete samples, and performed laboratory tests, using the results to construct ANN training vectors, to locate and identify steel bars under the concrete cover.

Ground penetrating radar (GPR) is the best known technique in the NDT area based on high-frequency electromagnetic fields. Annan [18] and Blindow [19] are good references to understand the principles of GPR. Farnoosh *et al*. [20] presented electromagnetic and computational aspects of the technique, using a numerical analysis. According to these authors, one of the biggest difficulties in the use of the GPR technique is that skilled personnel should analyze the results, and it involves a considerable amount of post-processing work. Shaw *et al*. [21] used GPR results to construct ANN training vectors to locate and identify steel bars in reinforced concrete structures.

Concerning corrosion assessment in the reinforcement of concrete structures, there are few works using GPR techniques. In [22] the authors describe laboratory experiments on the influence of moisture and chloride contents on the amplitude of radar signals. In reference [3], the authors used low-profile ultra-wide-band antennas and twelve concrete samples with induced corrosion, to correlate electromagnetic signatures with the corrosion level of the steel bars. The results were compared with numerical simulations, to verify their consistency.

Radiography is one of the earliest NDT techniques used for imaging the steel reinforcements immersed in the concrete. Due to their very small wavelengths, they propagate through the material along straight paths without any significant diffraction. X- and gamma-ray methods are capable of producing accurate two-dimensional images of the concrete interior. However, their use in concrete testing is limited, due to their high initial costs, relatively low speed, heavy and expensive equipment, need for extensive safety precautions and highly skilled operators, and perhaps most important of all, the requirement of accessing both sides of the structure (Buyukozturk [23]).

Thermography is another NDT technique based on electromagnetic principles used in the inspection of rebar-reinforced concrete structures. Baek *et al.* [24] proposed an integration of electromagnetic heat induction and infrared thermography to detect steel corrosion in concrete. They used an inductive heater to heat the steel rebar remotely from the concrete structure surface, integrated with an IR camera to capture the heat signatures.

## 3. Description and Basics of the Developed ECT Sensors for Steel Bar Inspections

### 3.1. Mathematical Basics

**Figure 2:**
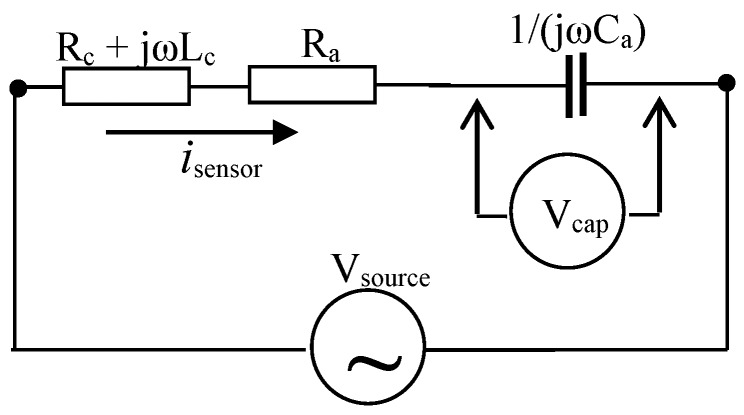
The electrical equivalent circuit of the ECT sensor for reinforcement inspections.

The sensors developed in this work use the eddy current principles. Their electromagnetic parts are, basically, RLC series circuits. An RLC series circuit is an association in series of a resistor, an inductor, and a capacitor. In this case, *L* is the inductance of a sensor coil, *L_c_*, *R* is the sum of the coil resistance *R_c_* and any other additional resistances *R_a_*, and *C* is the capacitance of an external capacitive array, *C_a_*, placed in series with the coil. Figure 2 shows an electrical equivalent circuit for the sensor.

The following simplifying assumptions limit the use of this equivalent circuit: (1) the capacitors do not present electrical resistances; in other words, only displacement currents are considered within the capacitors; (2) if external resistors are added to the sensor, they do not present both inductive and capacitive effects and only conduction current will be present in these resistors; (3) the operational frequency of the sensors will be in the range of 7.5–15 kHz, so no capacitive effects will be considered in the sensor coil; (4) the model does not consider parasitic capacitances; (5) if contact resistances are known, they can be added to the model.

In Figure 2, an input *V_source_*, with angular frequency *ω = 2πf*, is the voltage applied to the terminals of the RLC circuit, *V_cap_* is the voltage at the capacitive array terminals, and *i_sensor_* is the loop current in the sensor. Initially, the analysis of the electrical circuit of Figure 2 will be done for the no-load condition (no ferromagnetic material placed under the sensor). The second law of Kirchoff allows to express, for the voltage at the source terminals:

(1) $$V_{source}=\;\left(R_{c}+\;R_{a}\right)i_{sensor}+j\left(\omega L_{c}-\frac{1}{\omega C_{a}}\right)i_{sensor}$$

and for the current:

(2) $$i_{sensor}=\;\frac{V_{source}}{\left(R_{c}+\;R_{a}\right)+\;j\left(\omega L_{c}-\frac{1}{\omega C_{a}}\right)\;}$$

**Figure 3:**
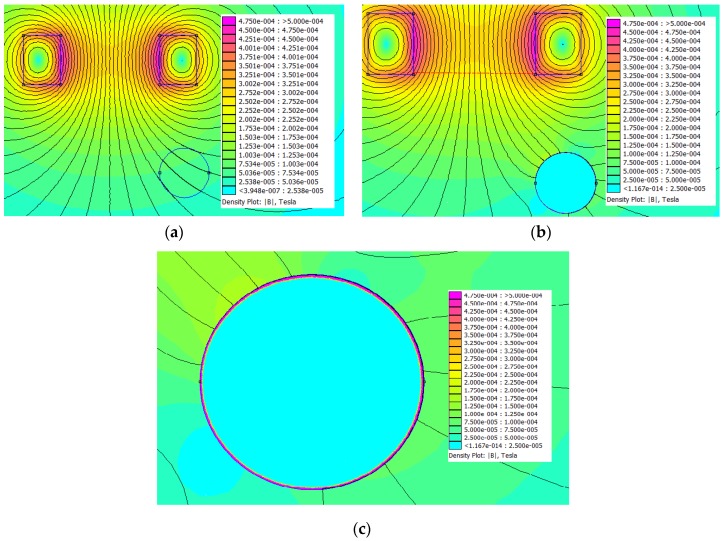
Simulation of the flux distribution under an ECT sensor. (**a**) Without the steel bar; (**b**) With the steel bar; (**c**) Details of the flux in the steel bar and region around it.

Before proceeding with the analysis of the equivalent circuit it is necessary to carry out an analysis of the behavior of the electromagnetic field in the region of interest, with the presence of a steel bar. As the sensor is fed by a time-varying voltage at its terminals, the resultant time-varying electromagnetic field will induce eddy current loops in the conducting body. The magnitude and behavior of the eddy current will depend on the magnetic flux density distribution, the metal conductivity, metal permeability, and the electrical frequency. The induced eddy currents will create a counter time-varying magnetic field that will disturb the original field. As an illustration, Figure 3 shows a field mapping for a 900 turn coil, fed by a voltage source of 5.0 V_rms_, with a frequency equal to 8.05 kHz. The non-commercial FEMM software [25] was used to perform the 2D simulations. Figure 3a shows the flux distribution in the region under the coil, without the presence of the steel bar. Figure 3b shows the flux distribution in the region under the coil with the presence of the steel bar, and Figure 3c shows details of the flux within the bar and in the region surrounding it.

Inspecting the maps of Figure 3, it is possible to see some expected phenomena from the electromagnetic theory: (1) the high permeability of the steel distorts the lines of flux around the bar; as is known, in a magnetic/non-magnetic interface, the lines of flux are perpendicular to the interface in the non-magnetic medium; (2) the high conductivity of steel does not permit the penetration of the magnetic flux within the bar; the flux density (and consequently the eddy currents) is confined to a tiny region around the interface between the bar and the surrounding non-conductor medium; in fact, the skin depth for the steel bar (calculated using the formula $\;\delta =1/\sqrt{\mu f\sigma }$), considering a relative permeability,$\;\mu_{r},$ equal to 1000, electric conductivity, σ, equal to 5.9 MS/m [16], and frequency f equal to 8.05 kHz is about 0.13 mm, is very consistent with the figures in Figure 3. Finally, magnitude and phase of the flux density are disturbed point by point. Table 1 shows the components of the magnetic induction vector at the center of the red line in Figure 3b.

**Table 1:** 2D components of the magnetic induction vector, with and without the steel bar.

	Bx (Wb/m^2^)	By (Wb/m^2^)
Without the bar	−3.665 × 10^−6^ − j6.346 × 10^−7^	2.526 × 10^−4^ − j1.224 × 10^−6^
With the bar	−4.240 × 10^−8^ − j5.172 × 10^−11^	2.522 × 10^−4^ + j1.815 × 10^−7^

As a result of the above discussion, the parameters of the electrical equivalent circuit will be affected in the following way: (1) ohmic losses will occur in the steel bar; this fact can be taken into account by adding a resistance ∆R_e_ in the equivalent circuit; (2) The coil inductance is no longer the original value, $L_{c}$. A new effective inductance $L_{ef}$ will be defined as:

(3) $$L_{ef}=L_{c}+\;\Delta L_{e}$$

where $\Delta L_{e}$ is a little change of the coil inductance, caused by the changes in the original magnetic field, by the presence of eddy currents in the steel bar.

The voltage at the sensor terminals will be now be expressed as:

(4) $$V_{source}=\;\left(R_{c}+R_{a}+\Delta R_{e}\right)i_{sensor}+j\left(\omega \left(L_{c}+\;\Delta L_{e}\right)+\frac{1}{j\omega C_{a}}\right)i_{sensor}$$

or:

(5) $$V_{source}=\;\left(R_{c}+R_{a}+\;\Delta R_{e}\right)i_{sensor}+j\left(\omega L_{c}-\frac{1}{\omega C_{a}}\right)i_{sensor}+j\omega \Delta L_{e}i_{sensor}$$

The sensor is designed to operate at its resonant frequency at no load. In other words, the inductive and capacitive reactances within the parentheses in Equation (5) will have the same value. or:

(6) $$\omega L_{c}=\frac{1}{\omega C_{a}}$$

and:

(7) $$f=\;\frac{\omega }{2\pi }=\;\frac{1}{2\pi \sqrt{L_{c}C_{a}}}$$

At the resonant frequency, the current in the sensor is:

(8) $$i_{sensor}=\;\frac{V_{source}}{\left(R_{c}+R_{a}+\;\Delta R_{e}\right)+j\omega \Delta L_{e}}$$

which after some algebraic operations are expressed as the sum of a real and an imaginary parts:

(9) $$i_{sensor}=\;\frac{V_{source}}{{\left(R_{c}+R_{a}+\;\Delta R_{e}\right)}^{2}+{\left(\omega \Delta L_{e}\right)}^{2}}\left(R_{c}+R_{a}+\;\Delta R_{e}-\;j\omega \Delta L_{e}\right)$$

The voltage at the capacitor (after some algebraic operations) is:

(10) $$V_{cap}=\;-\frac{V_{source}}{\omega C_{a}\left[{\left(R_{c}+R_{a}+\;\Delta R_{e}\right)}^{2}+{\left(\omega \Delta L_{e}\right)}^{2}\right]}\left[\omega \Delta L_{e}+\;j\left(R_{c}+R_{a}+\;\Delta R_{e}\right)\right]$$

The phase angle for the sensor current, $\phi_{c},$ is ${\mathrm{tg}}^{-1}(-\omega \Delta L_{e}/\left(R_{c}+R_{a}+\;\Delta R_{e}\right))$, and phase angle for the voltage at the capacitor, $\phi_{v},\;$ is $tg^{-1}(\left(R_{c}+R_{a}+\;\Delta R_{e}\right)/\omega \Delta L_{e})$. The phase angle for the relation $V_{cap}/i_{sensor}$will be always ϕ=−π/2.

At no-load condition, $\Delta L_{e}=\Delta R_{e}=$ 0, and Equations (9) and (10) become:

(11) $$i_{sensor}=\;\frac{V_{source}}{R_{c}+R_{a}}$$

and:

(12) $$V_{cap}=\;-j\frac{V_{source}}{\omega C_{a}\left(R_{c}+R_{a}\right)}$$

Equations (11) and (12) express the maximum values of the current at the sensor, and of the voltage at the probe capacitor, respectively. Connecting a potentiometer in series with the sensor coil, the no-load condition (values of $i_{sensor}\;$and $V_{cap}$ without the presence of a steel bar under the sensor) can be periodically calibrated.

### 3.2. The Frequency-Adjustment Method to Calculate the Effective Resistance and Inductance

The mathematical development presented in the previous subsection shows that the current in the sensor and the voltage drop at the terminals of the capacitive array depend on variations of both resistance and inductance. Therefore, for a better use of the results obtained, it is important to have a way to calculate the resistances and inductances explicitly, after the measurements.

From finite element simulations, the authors observed that variations on the effective inductances between the load and no-load condition are less than 0.5%. Including this variation in the calculation of a new resonant frequency, the frequency variation is less than 0.25%. Based on this fact, the authors propose a simple method to extract the resistance and inductance values from the measurements. After reaching the resonant frequency of the no-load condition, the sensor is placed on the steel bar, and the frequency is adjusted until the new resonant condition is attained:

(13) $$L_{c}+\;\Delta L_{e}=\frac{1}{{\left(2\pi f_{n}\right)}^{2}C_{a}}$$

and:

(14) $$\left(R_{c}+R_{a}+\;\Delta R_{e}\right)=\;\frac{V_{source}}{2\pi f_{n}C_{a}V_{cap}}$$

where *f_n_* is the new resonant frequency.

### 3.3. Finite Element Simulations and Experimental Comparisons for an ECT Sensor

Finite element analysis is a very interesting way to investigate the behavior of electromagnetic devices. Through it, a good understanding of the phenomena involved can be obtained, in addition to the mathematical modeling of the problem. Moreover, prototypes are built with more confidence, if the expected results for their operation can be accurately predicted.

**Figure 4:**
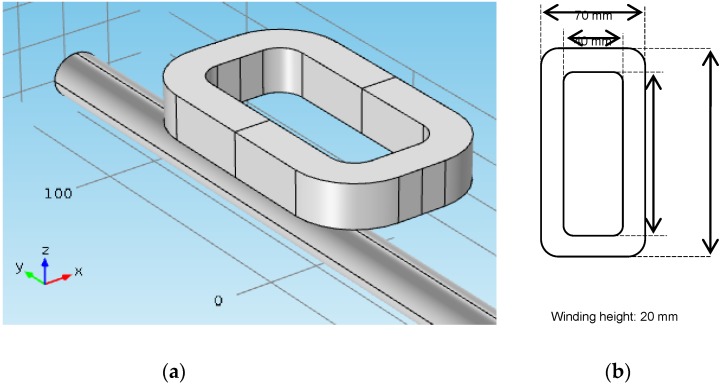
The electromagnetic components of an ECT sensor to inspect the reinforcement of concrete structures. (**a**) Perspective view; (**b**) Coil dimensions.

This subsection will present the construction details of an ECT sensor built from the mathematical model presented in the previous section. Figure 4 shows the electromagnetic component of this sensor. It is composed of a multi-turn coil with 900 turns of 24 AWG wire, connected in series with a capacitive array with capacitance equal to 5 nF and an additional resistance of 50 Ω (not shown in the figure for clarity). The dimensions of the coil are also provided in this figure.

**Figure 5:**
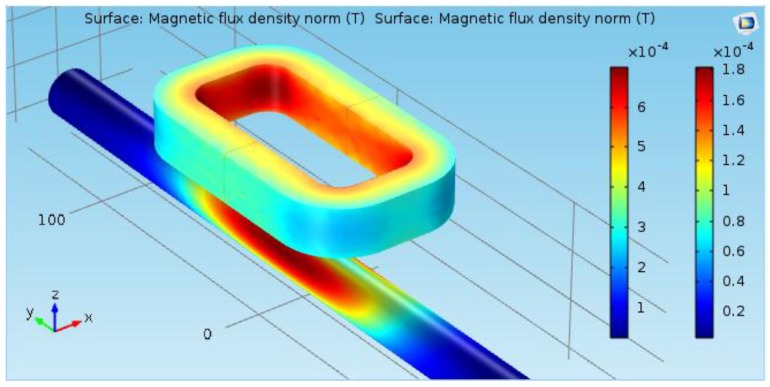
Magnetic flux density at the surface of the coil and the steel bar.

**Figure 6:**
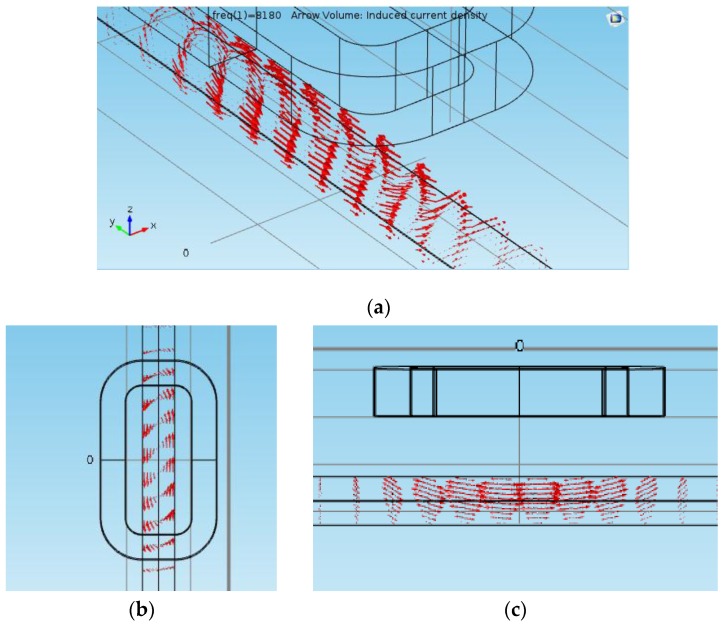
Induced current in the steel bar represented by the red arrows. (**a**) Perspective view; (**b**) Top view; (**c**) side view.

The authors performed 3D finite element frequency-domain simulations for this sensor using the commercial software COMSOL Multiphysics [26]. Figure 5 shows the magnetic flux density at the coil surface and the steel bar surface. The gauge of the bar is 20 mm, and the distance between the top of the bar and the sensor is 25 mm. Figure 6 shows the mapping of the eddy current induced in the steel bar. As can be seen from these pictures, the magnetic induction is very low elsewhere (magnetic saturation is not present), and the eddy currents are concentrated in the region of the steel bar right below of the sensor.

These figures are very interesting to understand the behavior of the field quantities involved. However, to evaluate if this sensor will produce the expected results other variables should be analyzed, such as the effective resistance and inductance of the winding, changes in the phase angle of the current in the sensor, and the voltage drop at the capacitive array. COMSOL Multiphysics was prepared to automatically perform simulations for two bar gauges, as well as for different positions of the steel bar in relation to the sensor.

**Figure 7:**
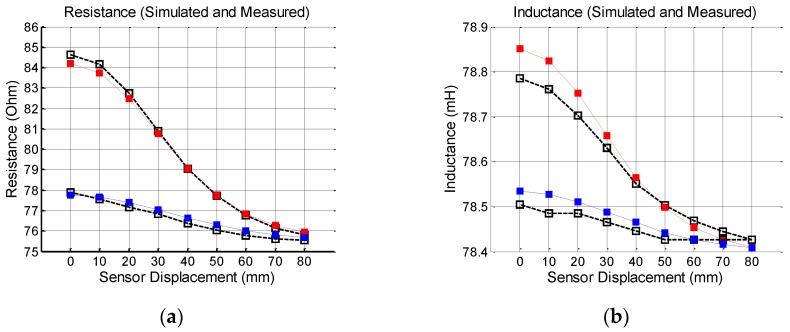

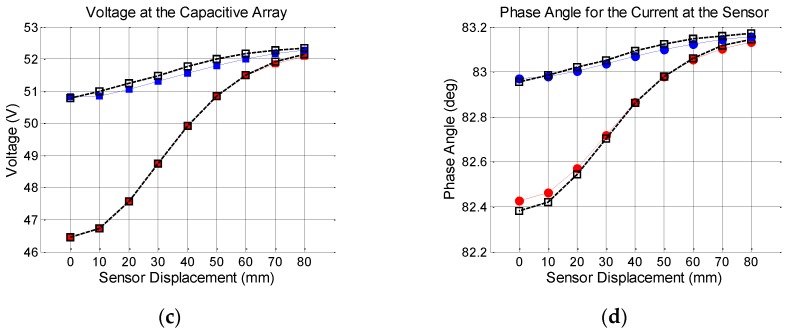
Simulated (color marks) and experimental results (hollow black marks) for the effective resistance (**a**); effective inductance (**b**); voltage (**c**); and phase angle (**d**) for a 20 mm steel bar. Red marks: Steel bar placed 25 mm under the sensor. Blue marks: Steel bar placed 45 mm under the sensor.

Simulations were done for a steel bar with gauge of 20.0 mm, placed at 25.0 and 45.0 mm under the sensor. Figure 7 show the results for the effective coil resistance, effective coil inductance, the voltage at the capacitive array and phase angle of the current in the sensor. The graphics also present the experimental values obtained for this sensor, but the methodology used for the experimental tests will be present in the subsequent sections. As can be seen, the simulated and experimental results agree very well each other.

## 4. The Production of Corroded Samples of Steel Bars

The corrosion process of the reinforcement of concrete structures is, in general, quite slow. Corrosion acceleration techniques are an important part of the studies on this subject. The impressed current technique is the most suitable one for this purpose. References [1,2,3,4,27,28] describe some research on this technique.

**Figure 8:**
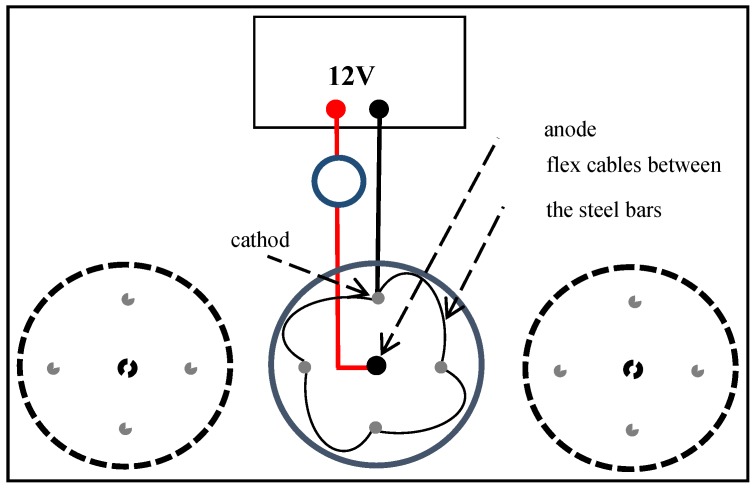
Schematic arrangement for the corrosion process.

**Figure 9:**
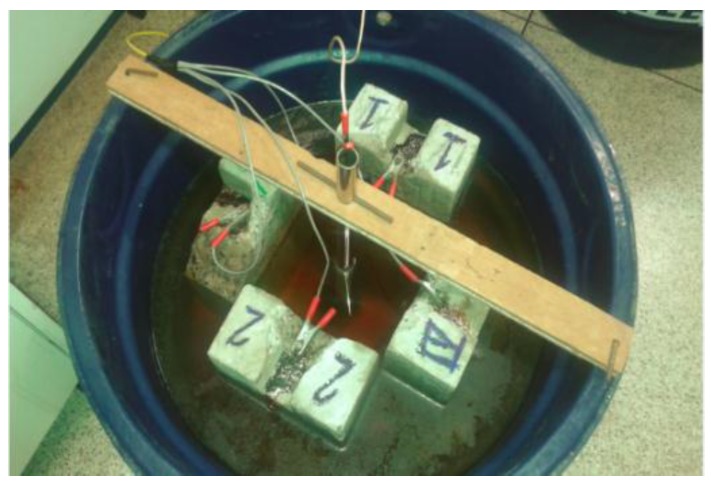
Four concrete samples in the process of corrosion, at the laboratory.

In this work, the impressed current technique was used to produce corroded samples of steel bars. Figure 8 shows the schematic arrangement of the experiment and Figure 9 shows four concrete samples in the laboratory during the corrosion process.

The purpose of the experiment was to obtain corroded samples of steel bars, with different levels of corrosion. In the context of this paper, corrosion level is correlated with the loss-of-mass of the steel sample. The concrete samples were immersed in a solution composed of 5 g of NaCl for each liter of water. A 12V DC battery was used to provide the electrical current. Care was taken to not allow the current in each tank to exceed 1.0 A, renewing the saline solution periodically. The concrete samples remained within the solution for periods between one and two months, to achieve different levels of corrosion. After the period of corrosion, the bars were removed from the concrete and the rust carefully cleaned. Finally, the bars were weighed, and their weight compared with the weight of samples of the same gauge and length, but not corroded. 

**Figure 10:**
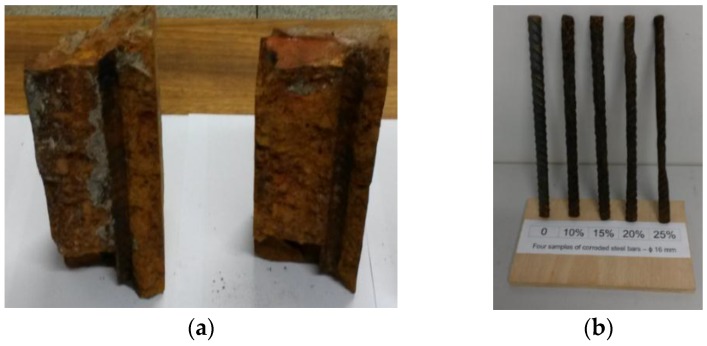
(**a**) Concrete debris; (**b**) Samples of corroded steel bars.

From the produced material, sixteen samples of corroded steel bars and four samples of non-corroded steel bars were chosen for this paper. The gauges used were: 10.0, 12.5, 16.0 and 20.0 mm. For each bar gauge, bars were chosen with corrosion levels close to 10%, 15%, 20% and 25%. Figure 10 shows the 16.0 mm gauge steel bar samples.

It should be pointed out that in the context of this work corrosion level is correlated with the loss-of-mass of the steel samples, but according to the simulations presented in the previous section, a more tighter correlation would be with the loss-of-cross-section-area of the bar, since the electro-magnetic field does not penetrate into the body of the samples.

## 5. Results

### 5.1. Experimental Setup

**Figure 11:**
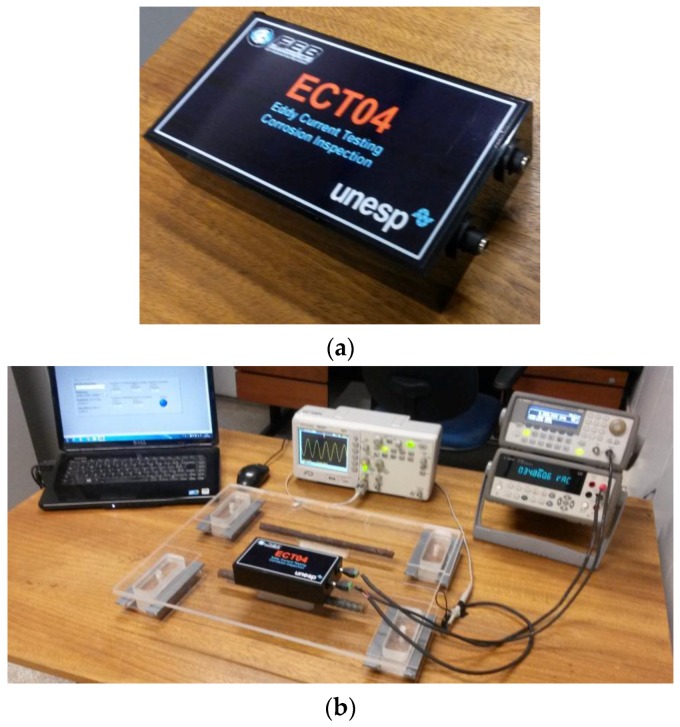
(**a**) An ECT sensor for corrosion inspection; (**b**) Experimental setup for the measurements.

For this paper, an ECT-RLC sensor, similar to those presented in Section 4, was built. The capacitive array is a 3 × 3 array of 5.0 nF capacitors connected series-parallel, resulting in an equivalent capacitance of 5.0 nF. The winding and the capacitive array were connected in series, inserted in a plastic box especially built for this, and after the internal connections, its interior was filled with a plastic resin. A U1733C handheld LCR meter (Agilent, São Paulo, Brazil) was used to measure the resistance, inductance and capacitance of the sensor at 10 kHz, and the measured values were: 73 Ω, 78.31 mH and 5.0 nF, respectively. Figure 11a shows a picture of the prototype used in the measurements. 

Figure 11b shows the experimental set-up used for this paper. It is composed of a signal generator to excite the probe at desired values of voltage and frequency, a digital desk-multimeter to measure the voltage at the capacitive array, an oscilloscope to inspect the quality of the signals and a laptop equipped with a LabView application to control all the equipment.

### 5.2. The Movement of the Sensor 

**Figure 12:**
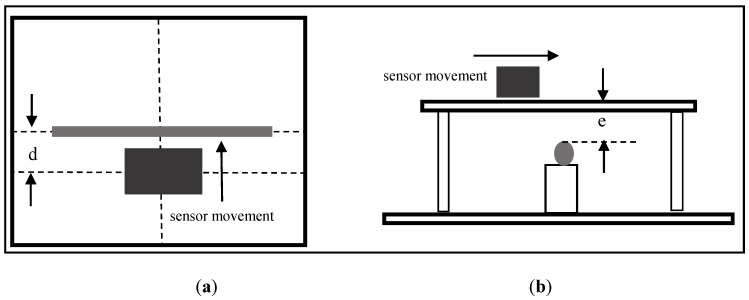
Schematic representation of the movement of the sensor: (**a**) top view; (**b**) side view.

The measurements were taken for two distances between the sensor and the bars (e = 25 and 45 mm). For the measurements already shown in Figure 7, the sensor was placed at nine positions (d = 0, 10, 20, 30, 40, 50, 60, 70, and 80 mm), in relation to the bar axis, as illustrated in Figure 12. The base frequency used in the test was 8043 Hz, the measured resonant frequency of the sensor.

### 5.3. The Procedure for the Measurements 

The procedure for the measurements was as follows:

- At no-load condition (no steel bar under the sensor):
  (1) The equipment is turned on, the resonant frequency is set in the signal generator and slightly changed until to reach the real resonant frequency for the sensor, 8043 Hz in this case. This frequency was used as starting frequency for all measurements with corroded or non-corroded steel bars, from this point on.
  (2) The coil inductance is calculated, using Equation (13).
  (3) The coil resistance is calculated, using Equation (14).
- At load condition:
  (4) A steel bar is placed under the sensor aligned with its main axis. The voltage at the capacitive array is measured and recorded. After this, the frequency slightly changed, up to the new resonance condition, and steps (2) and (3) are repeated, to calculate the effective resistance and effective inductance.
  (5) The phase angle of the current in the sensor is calculated using the extracted values of resistance and inductance.

**Figure 13:**
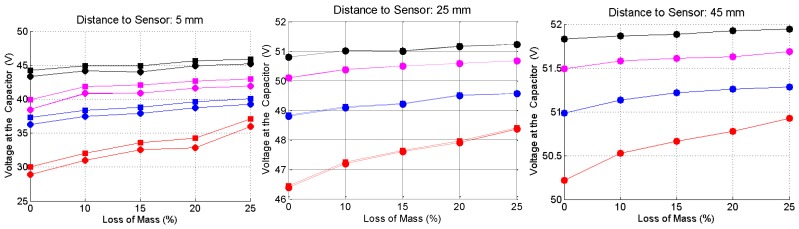
Measured voltage at the capacitive array for corroded (loss of mass equal zero) and non-corroded steel bars, as a function of the distance of the bar to the sensor. Red curves—20.0 mm bar gauge; Blue curves—16.0 mm bar gauge, magenta curves—12.5 mm bar gauge; and black curves—10.0 mm bar gauge.

Figure 13 shows the voltage at the capacitive arrays when the bars are placed at the reference levels of 5, 25 and 45 mm to the sensor. Circle marks are the results obtained before the adjustment of the frequency, and square marks are the results recorded after this adjustment.

**Figure 14:**
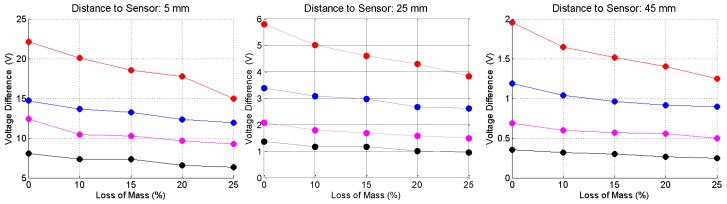
Voltage difference, as a function of the bar gauge, loss of mass and reference distance. Red curves—20 mm bar gauge; Blue curves—16.0 mm bar gauge; magenta curves—12.5 mm bar gauge; and black curves—10.0 mm bar gauge.

As can be seen, the differences between the values measured before and after the frequency adjustment become smaller, as the distance from the sensor increases. For a better understanding of the behavior of the sensor as a function of the bar gauge, corrosion level and distance from the bar to the sensor, Figure 14 shows the difference between the voltage at no load condition and the voltage with a steel bar under the sensor. As can be seen, these voltage differences are very well stratified, both in relation to the distance from the bar to the sensor, as in relation to the bar gauge. In a real field inspection, if the gauge of the bar is known, the corrosion level and the thickness of the concrete cover can be identified with enough confidence.

**Figure 15:**
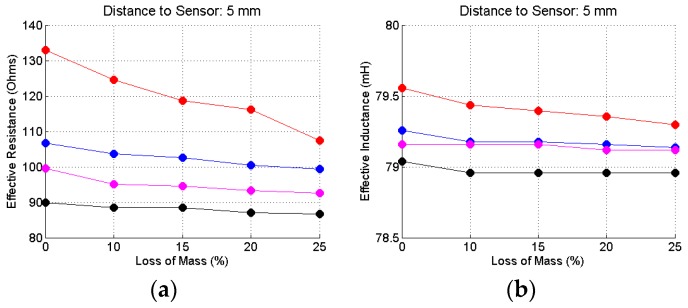
Resistance (**a**) and inductance (**b**) for the steel bars at the reference distance of 5 mm.

**Figure 16:**
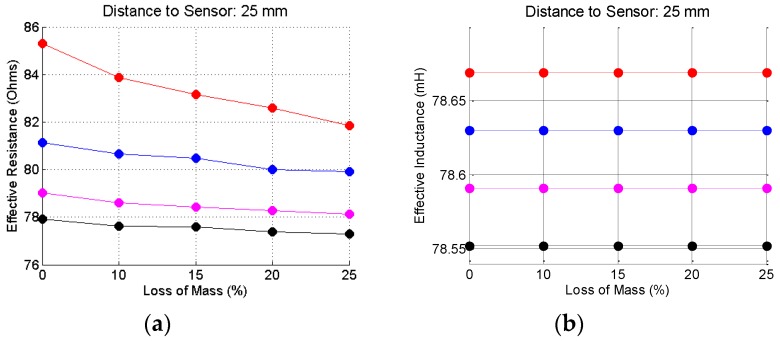
Resistance (**a**) and inductance (**b**) for the steel bars at the reference distance of 25 mm.

**Figure 17:**
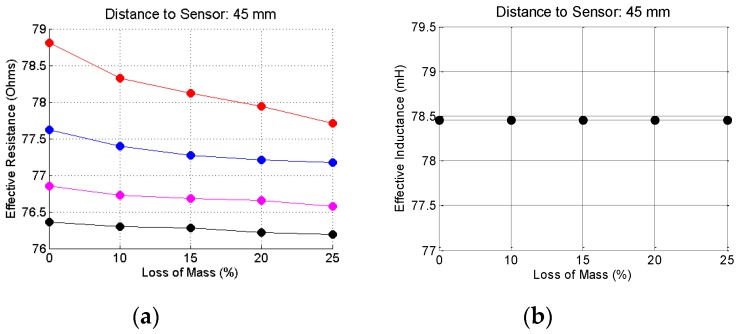
Resistance (**a**) and inductance (**b**) for the steel bars at the reference distance of 45 mm.

To complete this analysis, Figure 15, Figure 16 and Figure 17 show the effective resistances and inductances, calculated according to the procedure shown in the beginning of this section when the bars are placed at the reference distances of 5, 25 and 45 mm. The code color for these graphic is: red curves—20.0 mm bar gauge; blue curves—16.0 mm bar gauge; magenta—12.5 mm bar gauge; black curves—10.0 mm bar gauge.

Concerning the resistance, the variations are significant depending on: (1) the corrosion level; (2) the gauge of the steel bar and; (3) the distance of the bar to the sensor. Concerning the inductance, the variations are insignificant, depending on: (1) the corrosion level; (2) the gauge of the steel bar and (3) the distance of the bar to the sensor. The most significant changes occur for the distance of 5 mm. However, this would not be a usual distance between the bar and the sensor in a field test. The thickness of the concrete cover must be at least equal to the gauge of the bar, which does not happen in this case. With regard to the distance of 25 mm, there are slight changes, depending on the gauge of the bar, but that remains constant, regardless of the corrosion level. With regard to the distance of 45 mm, apparently there is no significant change in the inductance values.

As a conclusion of this analysis it is apparently sufficient to calculate the effective resistance, from the data obtained in the measurements, to develop a method for the inspection of reinforced concrete structures, both as regards the identification and location of the reinforcement, as for the detection of corrosion process of the steel bars.

### 5.4. The Lift-Off Effect

In eddy current NDT tests, “lift-off effects” are the effects caused by undesired variations of the distance between the sensor and the specimen, and can easily mask the test results. In this article, a fast investigation was conducted to evaluate the lift-off effect on the resistance and inductance values. The experiment to investigate the lift-off effects was done with non-corroded bars with a gauge of 20 and 16 mm. The bars were placed at the reference distance of 25 and 45 mm, and for each one, four lift-off values were used: 2, 4, 6 and 8 mm. The results were compared with those obtained for the original distance.

**Figure 18:**
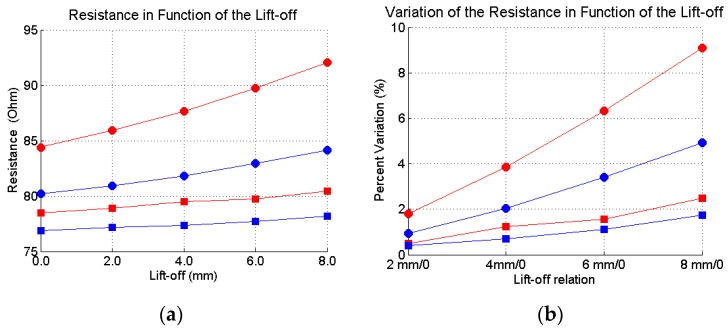
Lift-off effects on the resistance values. (**a**) absolute values; (**b**) Percent variation.

**Figure 19:**
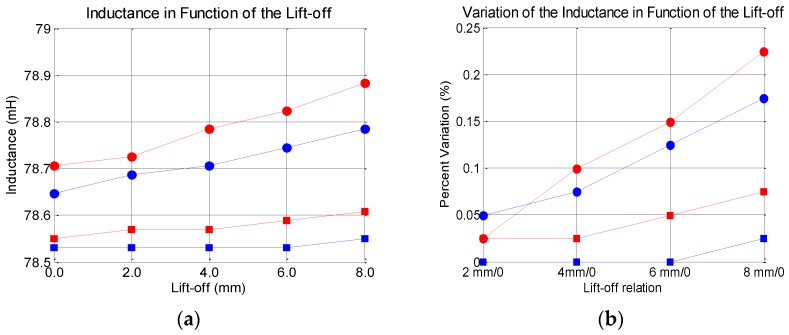
Lift-off effects on the inductance values. (**a**) absolute values; (**b**) Percent variation.

Figure 18 shows the effect of the lift-off on the resistance values, and Figure 19 shows the effect on the inductance values. Again, red curves stand for 20 mm bar gauge and blue curves stand for 16 mm bar gauge. Circle marks stand for 25 mm, and square marks stand for 45 mm between the sensor and the bar. As can be seen, the effects are greater for the resistance. However, in a real field inspection, if the measures are taken with the sensor performing small offsets in the axial direction, probably the lift-off should not affect the results substantially. However, this is an issue that must be carefully considered in the development of a real system for the inspection of concrete structures.

### 5.5. Discussion 

By analyzing these results, the conclusion is that the use of eddy current testing to identify the process of corrosion in the reinforcement of concrete structures can lead to a reasonable level of success. The experimental results presented here are the first approach of the authors, and of course, they can be improved. The sensor was taken to operate in its frequency of resonance and minor variations around it. The frequency-adjustment method proposed in the mathematical development was successful. In a general way, the results showed a consistent behavior, with the highest values being obtained for the highest levels of corrosion. Comparing the results on the gauge of the bars, was possible to perceive a logical sequence, with the lowest values for the gauge of 10 mm, and the highest values for the gauge of 20 mm.

A practical methodology for the use of ECT in the identification of corrosion processes in the reinforcement of concrete structures can be outlined as follows: first, measurements can be made over non-corroded parts of the reinforcement. In this way, the gauge and concrete cover (if not yet known) can be determined. After, measurements can be made successively along the reinforcement, comparing the results between then and with those for the non-corroded parts.

The way forward is now to conduct extensive laboratory and field measurements, to establish large datasets that can be used to feed artificial intelligent tools, like artificial neural networks or fuzzy logic, to develop expert systems for the detection of corrosion in the reinforcement of concrete structures.

## 6. Conclusions

A theoretical and experimental study was carried out for determining the corrosion level of steel bars used in reinforced concrete structures, using eddy current testing. The following steps were followed: theoretical review, with an overview of the main types of methods in the analysis of the corrosion of reinforcement of concrete structures; mathematical development of the circuit theory, to obtain expressions for the parameters and electrical variables of interest for the problem.; finite element simulations to understanding the electromagnetic phenomena involved in the analysis, and to predict the behavior of the parameters and electrical variables of the proposed sensors; experimental tests for the acceleration of the corrosion of steel bars in reinforced concrete structures; experimental measurements with the sensor using corroded and non-corroded samples; comparisons between simulated and experimental results; analysis of the results obtained for the corroded samples. All these steps were successful.

The methodology presented here is not a substitute for the well-established electrochemical methods already in use. It can be used as a preliminary assessment of the reinforcement of concrete structures in search of corrosion processes, and further work can be done, using other methods, to obtain a more comprehensive diagnosis of the problem. ECT sensors based on the principles presented here can be easily built, and operated by personnel with basic professional training, without the need for further knowledge in electrochemistry, for example. Finally, there is a wide field to be explored on this subject, as new levels of frequencies, multi-frequency sensors, improvement in the dimensions of the sensors, sensors with ferrite cores, *etc.,* are all topics worth pursuing.
